# Epistemic relativism, scepticism, pluralism

**DOI:** 10.1007/s11229-016-1041-0

**Published:** 2016-02-23

**Authors:** Martin Kusch

**Affiliations:** Department of Philosophy, University of Vienna, Universitätsstraße 7, 1010 Vienna, Austria

**Keywords:** Objectivity, Epistemic relativism, Epistemic systems, Pluralism

## Abstract

There are a number of debates that are relevant to questions concerning objectivity in science. One of the eldest, and still one of the most intensely fought, is the debate over epistemic relativism. —All forms of epistemic relativism commit themselves to the view that it is impossible to show in a neutral, non-question-begging, way that one “epistemic system”, that is, one interconnected set of epistemic standards, is epistemically superior to (all) others. I shall call this view “No-metajustification”. No-metajustification is commonly taken to deny the *objectivity* of standards. In this paper I shall discuss two currently popular attempts to attack “No-metajustification”. The first attempt attacks no-metajustification by challenging a particular strategy of arguing in its defence: this strategy involves the ancient Pyrrhonian “Problem of the Criterion”. The second attempt to refute No-metajustification targets its metaphysical underpinning: to wit, the claim that there are, or could be, several fundamentally different and irreconcilable epistemic systems. I shall call this assumption “Pluralism”. I shall address three questions with respect to these attempts to refute epistemic relativism by attacking no-metajustification: (i) Can the epistemic relativist rely on the Problem of the Criterion in support of No-metajustification? (ii) Is a combination of Chisholmian “particularism” (i.e. the insistence that we know lots of things) and epistemic naturalism an effective weapon against No-metajustification? And (iii) is Pluralism a defensible assumption?

## Introduction

There are a number of debates that are relevant for reflections on objectivity in science. One of the eldest, and still one of the most intensely fought, is the debate over epistemic relativism. Many philosophers think that if epistemic relativism is right then there is less to the objectivity of science than we commonly assume. This paper will not discuss this conditional. Instead it will seek to contribute to the question whether epistemic relativism is a defensible position. I hope that the relevance of my discussion for the guiding theme of this special issue will nevertheless be obvious throughout.

All forms of epistemic relativism commit themselves to the view that it is impossible to show in a neutral, non-question-begging, way that one “epistemic system”, that is, one interconnected set of epistemic standards, is epistemically superior to (all) others. Following Seidel (2014, p. 32) I shall call this view “No-metajustification”. No-metajustification is commonly taken to deny the *objectivity* of epistemic standards. In this paper I shall discuss two currently popular attempts to attack “No-metajustification”. The first attempt seeks to undermine No-metajustification by challenging a particular strategy of arguing in its defence: this strategy involves the ancient Pyrrhonian “Problem of the Criterion”. The second attempt to refute No-metajustification targets its metaphysical underpinnings: to wit, the claim that there are, or could be, fundamentally different and irreconcilable epistemic systems. Following Paul Boghossian I shall dub this assumption “Pluralism” (2006, p. 89).

I shall address three questions with respect to these attempts to refute epistemic relativism by attacking No-metajustification: (i) Can the epistemic relativist rely on the Problem of the Criterion in support of No-metajustification? (ii) Is a combination of Chisholmian “particularism” (i.e. the insistence that we know lots of things) and epistemic naturalism an effective weapon against No-metajustification? And (iii) Is Pluralism a defensible assumption? (i) and (ii) relate to the first form of attack mentioned in the last paragraph; (iii) relates to the second. My paper has three main parts, each one of which addresses one of my three key questions. I begin with my first key question, to wit, the question whether the epistemic relativist can use the Problem of the Criterion to make her case.

## Can the epistemic relativist use the problem of the criterion?

### Howard Sankey’s proposal

Luper (2004), Williams (2007) and Sankey (2010, 2011, 2012, 2013, 2014), forthcoming) maintain that the epistemic relativist’s best bet for a defence of No-metajustification is to rely on the “Problem of the Criterion”. Sankey (2011) offers textual evidence from well-known friends and foes of epistemic relativism: Sir Karl Popper, William Bartley, Thomas Kuhn, Paul Feyerabend, Barry Barnes, David Bloor, Larry Laudan, and John Worrall.

The starting point of the ancient Pyrrhonian Problem of the Criterion is the idea that the epistemic justification of beliefs presupposes epistemic criteria, that is, epistemic standards, norms, or principles (terms which I shall use interchangeably in what follows). The sceptic then points out that such standards too need to be epistemically justified. And finally the sceptic argues that any attempt to provide such epistemic justification of standards invariably ends up in an infinite regress, a circle, or a dogmatic termination. Or, in Sankey’s formulation:

If the criterion is justified by a further criterion, the sceptic requests justification of the further criterion in a manner that leads to an infinite regress. If appeal is again made to the original criterion, the justification proceeds in a circle and thereby fails to defend the original criterion. If the regress is halted by the adoption of a criterion without justification, the criterion fails to be adopted on a rational basis. (Sankey 2010, p. 5)

Since none of these three options is acceptable, or so the sceptic assumes, he proceeds to his conclusion: none of our standards is ever justified, and—consequently—neither is any of our beliefs.

According to Sankey, the relativist turns the sceptical *Problem* of the Criterion into the relativist so-called “*Argument* of the Criterion”. The epistemic relativist agrees with the sceptic concerning the first step of the Problem of the Criterion: that is, the view that no *standard* ever has an “objective, rational justification” (Sankey 2010, p. 1). There is no neutral epistemic ranking of epistemic systems or standards. The epistemic relativist refuses to take the second step, however. This is the step taking us from ‘no *standard* is ever epistemically justified’ to ‘no *belief* is ever epistemically justified’. The epistemic relativist holds that at least some beliefs are justifiable in terms of so-called “operative standards”. These are standards that we have adopted without a good epistemic reason:

... the decision to adopt a given epistemic norm is not one that may be made on a rational basis. Nor is it possible for any particular epistemic norm to receive greater justification than any other. For all norms are equally lacking in justification. Instead of being a rationally based decision, the adoption of a norm is rationally unjustified. It may rest upon an irrational leap of faith, a subjective personal commitment or an arbitrary convention. (Sankey 2010, p. 5)

From here the epistemic relativist proceeds to his claim that different (systems of) epistemic standards are equal: different sets of operative standards are all on one par in so far as they are all without epistemic justification. And thus the relativist takes himself to have defended No-metajustification.

### Four objections

Sankey’s rational reconstruction of the epistemic relativist’s use of the Problem of the Criterion can be challenged on at least four grounds. First, Seidel denies that the relativist can, as it were, “get off” the sceptical train of thought before reaching the full-blown sceptical conclusion. Seidel reasons as follows. The Problem of the Criterion undermines *every* kind of epistemic justification, not just its absolute form. And thus there is no space for a justification of beliefs in terms of operative standards (Seidel 2013a, p. 136).

A second worry has been voiced by Pritchard in a different context (Pritchard 2009, p. 400). If the relativist believes that his epistemic system is no better than any other, then how can he grant his beliefs *any* positive epistemic status? But if he cannot give his beliefs any positive epistemic status, then his position collapses into scepticism. Paul Boghossian makes essentially the same point when he asks how—after relativizing the property of epistemic justification to communities—the relativist can still treat epistemic justification as normative (2006, p. 91).

Third, Seidel laments that the relativist Argument of the Criterion does nothing to undermine epistemic absolutism. Epistemic absolutism is a *metaphysical* doctrine, to wit, the view that *there are* absolutely correct epistemic standards. And yet, the Problem or Argument of the Criterion establish no metaphysical conclusion. Sceptics and relativists themselves only take these considerations to give support to the *epistemological* thesis that it is impossible to justify epistemic standards (Seidel 2013b, p. 148).

Fourth, in fact Seidel thinks that the Argument of the Criterion achieves even less than showing that it is impossible to justify epistemic standards. The argument does not even block all *epistemic* resources of the absolutist. If successful the Argument of the Criterion shows that we cannot “justify that a *particular* norm has a higher epistemic status than alternative epistemic norms”. And yet, the Argument of the Criterion does not disprove “the claim that we are justified to think that *there are* absolutely correct epistemic norms”. The absolutist might establish the latter claim, Seidel thinks, by showing that epistemic relativism is self-refuting (Seidel 2013b, p. 149).

### A sketch of a defence of the argument from the criterion

I shall now turn to assessing these objections. The first thing to note is that Sankey and Seidel portray the debate between epistemic relativist and her opponent as one between two camps of epistemic internalists committed to the view (the “JJ-principle”) that if a subject S is epistemically justified in believing that p, then S is justified in believing that S is justified in believing that p. Of course many epistemologists are externalists, and many epistemic internalists reject iteration principles (cf. Alston 1986, 1993; Goldman 1999; Bergmann 2004; Sosa 1994, 1997). In what follows I shall stick to Sankey’s and Seidel’s way of framing the debate, leaving a discussion of No-metajustification in light of externalism and iteration-denying internalism to another occasion. Moreover, what I shall offer here are no more than sketches of strategies for defending the Argument from the Criterion. To fully develop these strategies would demand much more space than I have here.

I begin with a comment on what damage the Problem of the Criterion does to epistemic absolutism. I agree with Seidel that the epistemic relativist needs an independent argument against the self-refutation charge, an argument that differs from the Argument of the Criterion. (I am optimistic on this score, but this is a topic for another paper.) Seidel is also right to say that the Argument of the Criterion—as an *epistemological* argument—cannot by itself establish the *metaphysical* conclusion that there are no absolute epistemic standards. And yet, while all this is true, it is hard to disagree with Paul Boghossian—certainly no friend of relativism—when he writes that: “If there are absolute epistemic facts about what justifies what, then it ought to be possible to arrive at justified beliefs about them” (2006, p. 74). At least this is so, Boghossian maintains, as far as rough approximations of absolute epistemic facts are concerned (2006, p. 76). Luper—another critic of relativism and scepticism—agrees and calls this view a “form of verificationism” (2004, p. 277). An absolutist who denies Boghossian’s and Luper’s intuitions, it seems to me, is actually in danger of falling into scepticism himself. Or, as Boghossian puts it: “... what would be the interest of an absolutism about epistemic truths which combined that absolutism with the affirmation that those truths are necessarily inaccessible to us?” (2006, p. 76). If Boghossian and Luper are right, however, then the Argument of the Criterion cannot be dismissed in Seidel’s fashion. By the absolutist’s own standards, the relativist can argue as follows: if it is impossible to arrive at justified beliefs about so-called “absolute epistemic facts” then we have reason to think that there are no such facts.

What can be said to answer the charge that the epistemic relativist—in using the Problem of the Criterion—is committed to going all the way to scepticism? As we saw above, Sankey (2013) seeks to answer this concern by suggesting a sharp distinction between the justification *of standards* and the justification *of beliefs in terms of standards*. The Argument of the Criterion undermines the former, but not the latter. I have two worries about this answer. The first is that it is hard to find any card-carrying modern-day relativist who actually defends such a view. (And remember that Sankey sets out to reconstruct “real” relativists’ use of the Problem of the Criterion.) My second concern derives from the increasingly influential “hinge epistemology”, that is, the strand of epistemology following the leads of Ludwig Wittgenstein’s *On Certainty* (Wittgenstein 1969; Coliva 2010, 2015; Moyal-Sharrock 2007; Pritchard 2015; Wright 2004). It is a widely shared assumption in this literature that Wittgensteinian “hinge propositions” or “certainties” are beliefs and epistemic standards *at the same time*. If that is correct—and I do not have space to argue for it here—then Sankey’s strict division between beliefs and standards cannot stand.

Sankey’s strategy for blocking Seidel’s first criticism thus does not fully convince. Is there perhaps a better strategy? I only see one challenging but perhaps not altogether hopeless alternative. The epistemic relativist might motivate her position as a *diagnostic form of anti-scepticism*. Assume the Pyrrhonian sceptic is able to convince us that we cannot epistemically justify our epistemic standards in ways that avoid infinite chains, circular reasoning, and contingent, local, and variable starting points. For the absolutist this is a devastating result. This is because, for the absolutist, absolutely correct epistemic standards can in principle be *shown to be* absolutely correct or justified. And here absolute epistemic justification is thought to be incompatible with infinite chains, circularity or contingent starting points. Hence the absolutist has to accept the sceptical conclusion: we can never have epistemically justified beliefs. The relativist’s response to the Problem of the Criterion is different. The relativist takes the sceptical result to show that epistemic absolutism is wrong since it is unable to defend our deeply held pedestrian (“particularist”) conviction that we know lots of things. To defend this pedestrian conviction, the relativist maintains, we need to reject the idea of absolutely correct epistemic standards with their absolute epistemic justifications. We can rescue our pedestrian conviction if we demand less of our epistemic standards and less of epistemic justification. We demand less of our epistemic standards if we allow them to vary with communities, and if we accept that they cannot be ranked in a neutral way. We ask less of epistemic justification if we allow that it involves infinite chains, circularity and contingency. In this spirit, the epistemic relativist might suggest that the epistemic justification of one’s epistemic standards will invariably have a strong element of circularity: to evaluate our standards we have to rely on our standards. And there is no way to avoid contingency either: what epistemic system one finds oneself with is determined by circumstances beyond one’s control.

Of course, lowering our expectations concerning what epistemic justification amounts to is not yet to embrace the idea that there are incompatible though equally valid epistemic systems. After all, there is also *non-relativist* diagnostic anti-scepticism. That is to say, faced with the Problem of the Criterion, many epistemic absolutists adopt the general idea of diagnostic anti-scepticism *but without* the relativistic conclusion. In other words, this brand of absolutists agrees that some of the core assumptions about epistemic justification have to be modified if scepticism is to be blocked. And yet, these absolutists insist that such revisionism does not lead to relativism. After all, “infinitism”, “foundationalism”, “coherence theory” and “reliabilism” are all candidates for revisionist forms of epistemology that block the Problem of the Criterion. The infinitist allows for infinite chains of justification; the foundationalist accepts endpoint of justification; the coherence theory leaves room for certain types of justificatory circularity; and the reliabilist goes beyond the internal perspective of the epistemic subject.

If epistemic relativism is to be convincing, then it must be able to show that all of these positions are plausible only in a relativistic garb. This is a tall order. But it does not strike me as altogether hopeless. After all, there are plenty of criticisms of these positions in the literature already, and some of these criticisms might well play into the relativist’s hand. (Think, e.g., of Robert Brandom’s criticisms of reliabilism; Brandom 2001). Note however that if this train of thought is near the mark, then the Argument of the Criterion is no shortcut to epistemic relativism. The relativist has to face the hard and tedious work of criticising and interpreting specific non-relativist epistemic proposals on epistemic justification.

Assume that the epistemic relativist could deliver on these promises. Would that be enough to escape scepticism? As I mentioned above, Pritchard and Boghossian do not think so: if I take all epistemic systems to be equally valid, what grounds do I have for thinking that my beliefs have any positive epistemic status at all?

In order to answer this challenge, it seems to me, the relativist must formulate his position in a way that involves two perspectives. The first perspective is one we happen to have because of contingent historical circumstances. We find ourselves ‘thrown’ into historically contingent epistemic communities with their historically contingent standards and beliefs. We have our epistemic system through our training and education. And on the basis of our training and education, our standards appear or *seem* to us compelling and without alternative. We do not have such *seemings* for epistemic systems other than our own. We take it that these seemings give us some justification for privileging our own epistemic system: just like perceptual seemings justify perceptual beliefs, so intellectual seemings justify at least some epistemic justification for our epistemic systems (Rosen 2001). It is for this reason that intellectual seemings are more than just features of familiarity. Further justification for privileging one’s own system might invoke an idea central in Boghossian‘s arguments *against* relativism: that is, the idea that we are blindly entitled to our home system. Boghossian characterizes the idea as follows (2006, p. 99): “... each thinker is entitled to use the epistemic system he finds himself with, without first having to supply an antecedent justification for the claim that it is the correct system.”

When we encounter an alternative epistemic system, we will often be inclined to accept that *if only* we had been raised in that system, it might well have seemed right to us. In some cases this insight leads us to weaken our commitment to our own epistemic system. In other cases it does not (Rosen 2001). Sometimes we decide to switch to the other system, sometimes we reject it, and sometimes we move in a sceptical direction. In the last-mentioned case the very fact of disagreement makes us doubt the possibility of knowledge or justified beliefs in the given area. All these are *non-relativist* responses to an encounter with another epistemic system. The *relativist* response is to think that although the beliefs demanded or licenced by the other epistemic system do not seem right to us in light of our own epistemic system, they are nevertheless justified given the other epistemic system. Moreover, there is no way to show in a neutral way that our system is superior. And finally, the other system seems as correct to its users as our system seems to us.

This brings us to the second perspective. The second perspective is based on reflection about one’s epistemic practices or standards. To be an epistemic relativist is to reflect on the contingency of one’s beliefs and standards, as well as their historical variability, and to conclude that one’s own position lacks a special privilege as compared with others. When taking this second perspective, we—as it were—“step outside” of the role of epistemic agents, and see ourselves from the perspective of the sociologist or anthropologist. Note however that taking this second perspective does not mean abandoning the first altogether. From the first perspective our epistemic standards continue to strike us as right.

The relativist thinks that going back and forth between these two perspectives—“meta-alternation” as relativist sociologists call it (Collins and Yearley 1992, p. 301)—is inevitable. Moreover, the two perspectives can of course yield different verdicts with respect to the same question. The absolutist will interpret this as a refutation of the relativist’s position. For the relativist, the fact that the two perspectives may yield different verdicts on the very same question, does not constitute a refutation. Different verdicts from the two perspectives can be dealt with by exercising judgement and finding compromises. There is indeed a lack of coherence here—if we take the two perspectives as *one* body of beliefs—but the demands of coherence need not apply across the perspectives. To demand coherence across perspectives is to set too high a standard of coherence, insists the relativist.

But how can the rational relativist hold both perspectives at the same time? How does holding the first and second (i.e. second-order) perspectives differ from holding two incompatible first-order perspectives? Why should the enlightened relativist not dismiss her intellectual seemings are mere signs of irrational attachment, familiarity or homeliness? And do not intellectual seeming obviate the need to justify one’s epistemic principles? Let me take these questions in turn.

The relativist can rationally adopt both perspectives since she is both an epistemic *agent* and an epistemic *analyst*. As an epistemic agent she forms beliefs and judgements using her epistemic system, a system that seems right and proper to her. The fact that it seems right to her makes it rational for her to use it. As an epistemic analyst she recognizes the contingency of her adhering to her epistemic system, and the rationally permissibility—by her standards—of other epistemic systems incompatible with her own.

Having both a first-order and a relativist second-order perspective is unlike possessing two incompatible first-order perspectives. Two incompatible first-order perspectives produce epistemic beliefs or judgements that contradict one another. The situation is different for the combination of first-order and relativist second-order perspectives. The second-order perspective does not have epistemic principles that directly compete with those of the first-order perspective. It regulates which epistemic systems are rationally permissible and as such equally valid. To say that an alternative system is rationally permissible is not to conclude that its principles are binding for me. It is rationally permissible for those who have the appropriate intellectual seemings. And these I just do not have.

But why shouldn’t the enlightened relativist dismiss her intellectual seemings as mere signs of irrational attachment, familiarity or homeliness? Our intellectual seemings are what give meaning and point to our cognitive projects. Our cognitive projects would cease to be cognitive projects for us if we came to see the seemings are mere irrational attachments or signs of mere familiarity or homeliness. This makes it rational for us to treat them as epistemic (cf. Wright 2014).

Finally, do intellectual seemings obviate the need for justifying one’s epistemic principles? Do they undermine the Argument from the Criterion? Here we need to distinguish between the epistemic agent (as construed by the relativist) and the absolutist epistemologist. The former is entitled for the most part to act on her epistemic seemings. The absolutist epistemologist—at least the absolutist epistemologist committed to internalism and the JJ-principle— insists that we can and must do better. It is he who is targeted by the Argument from the Criterion, not the “ordinary” epistemic agent.

I am not aware of anyone who has formulated such dual-perspective view for epistemic relativism. But Wong has defended such position for what he calls “moral pluralistic relativism” (2006). Wong is well versed in the Chinese philosophical tradition, and this enables him to trace this conception back to the fourth-century-BC Daoist philosopher Zhuang Zhou. The tension between the two perspectives—Wong calls them the “engaged” and the “detached” perspectives—is reduced by Zhuang and Wong insofar as the detached perspective can feed into the engaged perspective: seeing one’s own morality as one of many can be a way to prepare oneself for learning from other systems of morality—despite the overall relativistic outlook (2006, p. 235).

I am far from believing that the considerations developed in this section make a conclusive case for using the Problem of the Criterion in an effort to motivate epistemic relativism. But I hope to have said enough to enable the reader to judge the plausibility of my general argumentative strategy.

## Particularist naturalism as a strategy against epistemic relativism

### Particularist naturalism and the argument from the criterion

I now turn to the second of my three guiding questions: Is a combination of Chisholmian “particularism” (i.e. the insistence that we know lots of things) and epistemic naturalism an effective weapon against No-metajustification?

Chisholm asks epistemologists to “... distinguish [...] two pairs of questions ... (A) ‘What is the *extent* of our knowledge?’ and (B) ‘What are the *criteria* of knowledge?”’ (1973, p. 12). According to Chisholm, there are three philosophical responses to these questions. The sceptic insists that answers to A and B presuppose each other. Hence we cannot answer either question (1973, p. 14). “Methodists” assume they have a response to B and then reply to A on this basis. And “Particularists” claim to have an answer to A and then solve B using their reply to A. In other words, the particularists start from “the fact that we *do* know many things” (1973, p. 15). According to Chisholm, “... the third possibility (i.e. particularism) is the most reasonable.” At the same time however, Chisholm admits to not having a non-question-begging argument for particularism (1973, p. 21).

Chisholm’s work is important in our context since Sankey wishes to “combine a particularist stance with the naturalistic view that epistemic norms are subject to empirical evaluation” (2010, p. 8). The idea is that we test epistemic standards or norms against our existing knowledge, knowledge that—in line with particularism— we simply take for granted. If we follow this procedure, Sankey claims, then some epistemic norms turn out to be *objectively* better justified than others. And this disproves epistemic relativism as Sankey thinks of it. Consider, for instance, the famous Azande “Poison Oracle” (Evans-Pritchard 1976). Poison is administered to a chicken, and a yes-no question is addressed to the poison at the same time. The two outcomes—the chicken dies, the chicken lives—are linked to the “yes” and “no” options before the poison is forced down the poor chicken’s throat. Boghossian formulates the underlying epistemic principle as follows:

(Oracle) For certain propositions p, believing p is prima facie justified if a Poison Oracle says that p. (2006, p. 71)

Sankey thinks we can test this principle for reliability by checking its success rate in cases for which we have independent strong evidence concerning outcomes (say, the weather tomorrow). Sankey is also confident that the Poison Oracle will perform poorly in this test.

### A defence of epistemic relativism against particularist naturalism

I am not convinced. Take particularism first. Whether particularism is a promising strategy against epistemic scepticism is of course a very big issue—too big an issue to be decided here. I shall therefore set this question to one side and focus instead on the question whether particularism is an intuitively promising strategy against epistemic relativism.

It seems to me that particularism is not a promising strategy against epistemic relativism. Particularism is a natural (prima facie) response to radical scepticism, but it is much less intuitive as a response to relativism. Particularism is the expression of the belief that we *do* know many things. This contradicts the sceptic, but it does not contradict the epistemic relativist. The latter agrees—as long as the knowledge is relativized to epistemic systems.

It might be thought that the relativist’s commitment to particularism undermines the philosophical significance of No-metajustification. If it is both true that we have mundane knowledge and that our epistemic norms cannot be justified, then knowledge and justification do not presuppose metajustification. This worry rests on misunderstanding the dialectical situation. The Argument from the Criterion is directed at the absolutist internalist epistemologist who endorses the JJ-principle and thinks that we can do better than rely on particularism.

Note moreover that particularism is actually a family of views. Different family members disagree over how much knowledge they assume us to have. Chisholm does not restrict the relevant domain in any way. Moore’s particularism—and both Chisholm and Sankey refer to Moore as one of their ilk (Chisholm 1973, p. 22, Sankey, unpub., p. 19)—concerns a *philosophically reconstructed* set of common-sense beliefs. That is to say, many of the items of his list would sound a little awkward to the common man. And thirdly, there is what in everyday life we would refer to as “common sense (knowledge)”. This category consists of a mixed bag of platitudes and proverbs.

All three positions run into problems once we read them as particularist claims to *absolutist* knowledge. Chisholm’s particularism would then simply be the denial that knowledge can be relative. But again, this is far from obviously true. The everyday conception has to reckon with the existence of relativist anthropologists like Clifford Geertz who study “Common Sense as a Cultural System”. Geertz writes: “Common sense is not what the mind cleared of cant spontaneously apprehends; it is what the mind filled with presuppositions ... concludes” (1983, p. 84). There thus is not one common sense, there are many. And that hardly is grist to the mill of the anti-relativist. Finally, if we opt for Moore’s particularism, we have the Wittgenstein of *On Certainty* take over the part of Geertz. Wittgenstein gives many examples of tribesmen who do not share our, or Moore’s, certainties.

This is not to deny that Wittgenstein allows for the possibility that some certainties may well be shared by all of us, in all cultures. Coliva, Moyal-Sharrock and Pritchard regard this insight as a major blow to relativism (Coliva 2010, pp. 188–203; Moyal-Sharrock 2007, p. 147; Pritchard 2009). I disagree. Wittgenstein’s insight causes damage only to the most radical and implausible forms relativisms—that no-one has ever actually advocated. The sensible relativist is happy to acknowledge that there is a common core of abilities and beliefs (Barnes 1976), insisting at the same time that such common core leaves plenty of room for relativism. The relativist maintains that the common core plus the empirical input from the natural world *underdetermines* what we come to believe and know.

Turning to problems for the naturalistic testing of epistemic norms, we should once more remember that a sensible relativism need not deny that *some standards* can be epistemically justified. But the sensible relativist insists that such justification is dependent upon *specific* epistemic systems. And thus the question becomes: which system should be used? Different epistemic systems might interpret the tests for reliability in different ways—depending on their respective goals and values. They thus might focus on different reference classes, demand different size data, different numbers of test runs, a focus on either false positives or false negatives, and much else besides. In other words, a naturalistic testing of epistemic norms might well lead us back to epistemic relativism. (Indeed, the argument for epistemic relativism in good part rests on the fact that we find such pluralism of principles as soon as we start looking—say, in the history of science).

These considerations naturally apply to Sankey’s take on the Poison Oracle and its testability. Sankey assumes that there is a straightforward way to test the oracle’s reliability: we compare its predictions about the weather, for example, with the actual meteorological data. And given that the oracle does not do well by these standards, we have reason to reject the Oracle norm. This may well be right, by our (first-order) epistemic system, and by our rendering of the practice of the Azande. But it may well not be correct by the standards of the Azande, and by how they understand their use of the Poison Oracle. They are not using the Poison Oracle exclusively as a kind of weather report; they also use it as a device to resolve low-level conflict (e.g., Leeson 2014). (I feel slighted by you; I accuse you of acting as a witch towards me; the oracle says you are a witch; you make amends; and reaffirm your benevolence towards me). Given how well the Oracle traditionally does in this role, the Azande are likely to judge the Poison Oracle as reliable even if its metereological credentials are no better than a random number generator. To evaluate the Poison Oracle exclusively in the latter domain is a thought that is foreign to them, and odd given their epistemic system.

The point generalizes: epistemic testing of alternative principles is always possible. But it is possible only on the basis of some epistemic system or other. And there might be no perspective from which one such epistemic system is absolutely correct.

Summa summarum: I do not think that particularist naturalism is a stick with which to beat the relativist.

## Are there fundamentally different epistemic system?

### The critique of pluralism

I finally turn to my third question: Is Pluralism a defensible assumption? The relativist accepts Pluralism: there are, or could be, many fundamentally different and irreconcilable epistemic systems. Boghossian and Seidel challenge this view. Seidel follows Boghossian’s lead but tries to make his ally’s main arguments more precise. Both authors assume that for two epistemic systems to differ fundamentally, they must differ with respect to at least one fundamental (not derived) epistemic principle (Boghossian 2006, p. 69; Seidel 2014, p. 166). Boghossian and Seidel seek to show that there are no cases of such differences, or at least that none has been presented convincingly.

Seidel formulates two criteria meant to establish this thesis. The first he calls “Instance”. Assume we have one epistemic system, ES$_{1}$, with norm N’; and another epistemic system, ES$_{2}$, with norm N”. Allow further that N’ and N” are instances of a further norm N that is part of both ES$_{1}$ and ES$_{2}$. In such situation Seidel thinks we would all find it intuitive that ES$_{1}$ and ES$_{2}$ are not fundamentally different epistemic systems, at least not in virtue of their differing with respect to N’ and N”. Seidel explains and justifies the principle with the following example: ES$_{1}$ is the epistemic system of Platonism; ES$_{2}$ is the epistemic system of Aristotelianism; N’ is: “If Plato says p, then I am prima facie justified in believing that p”; N” is “If Aristotle says p, then I am prima facie justified in believing that p”. N’ and N” are instances of N: “If an ancient philosopher says p, then I am prima facie justified in believing that p.” Belief B’, occurring in ES$_{1}$ but not in ES$_{2}$, is: “Plato is an ancient philosopher”; and B”, occurring in ES$_{2}$ but not in ES$_{1}$, is : “Aristotle is an ancient philosopher.” Seidel’s verdict: However different the Platonists’ and the Aristotelians’ beliefs or derived norms may be, “we would not say” that they have “fundamentally different epistemic systems” (Seidel 2014, p. 169).

Seidel’s second principle is called “Derive”. Assume we have again ES$_{1}$ with norm N’ and ES$_{2}$ with norm N”. This time N’ and N” can both be derived from a further norm N that is part of both ES$_{1}$ and ES$_{2}$. Here too Seidel is confident that we would deem it intuitive that ES$_{1}$ and ES$_{2}$ are not fundamentally different epistemic systems, at least not in virtue of their differing with respect to N’ and N”. Seidel uses the same example as before except that N is now: “If an epistemologist says that p, then I am prima facie justified in believing that p.” B’ is: “An epistemologist told me that: ‘If Plato says p, then I am prima facie justified in believing that p.”’ And B” mutatis mutandis for the Aristotle. Seidel maintains that we have here no “fundamentally different epistemic systems.” We have different beliefs, different derived norms, but the same fundamental norms.

Following in Boghossian’s footsteps, Seidel applies these principles to the epistemic system of Cardinal Bellarmine (Galileo’s famous opponent in the Vatican). The idea is to check the thesis that Bellarmine had an epistemic system that differs from Galileo’s and ours. Boghossian suggests that Bellarmine’s epistemic system contains the following epistemic principle:

(Revelation) For certain propositions p, including propositions about the heavens, believing p is prima facie justified if p is the revealed word of God as claimed by the Bible (Boghossian 2006, p. 69).

Seidel goes on to claim that Revelation is justified by more fundamental epistemic principles, say about perception, deduction and induction. After all, Bellarmine used his eyes to read the Bible; he inferred propositions from what he read; and he assumed that the text of the Bible does not change from day to day. The use of these principles suggests to Seidel that Revelation must have been a derived principle (2014, p. 176; cf. Boghossian 2006, p. 103).

Seidel seeks to make a case for this conclusion also via a different route. For this purpose he contrasts Revelation with his own, alternative principle “Science”:

(Science) For certain propositions p, including propositions about the heavens, believing p is prima facie justified if p is included in the best physics books available (Seidel 2014, p. 175).

Seidel suggests that Revelation and Science are both “instances of more general norms regarding the reliability of books” (2014, p. 177). And this again shows that Revelation is not fundamental.

### A defence of pluralism

I have more than one concern about these arguments. Most of my objections take issue with what I regard as Boghossian’s and Seidel’s static and crystalline conception of epistemic systems. But I begin with an objection that grants the two authors this conception.

Consider an epistemic principle I propose calling “Mystical Perception”:

(Mystical Perception): If it seems to S that God is telling him that p; and if S is not already fully committed to atheism; and if circumstantial conditions D obtain, then S is prima facie justified in believing that God is telling him that p.

Mystical Perception is not part of my epistemic system but it is a fundamental principle in the epistemic systems of others. And at least some of these others cannot be easily dismissed as fools or religious fanatics. After all, the most detailed defence of the principle of mystical perception comes from the pen of the distinguished epistemologist William Alston, who wrote almost four-hundred pages on this topic (Alston 1991). Amongst other things, Alston argues in great detail that mystical perception has parallels with sensory perception in that neither have noncircular demonstrations of their reliability; both are self-supporting; both have overrider systems; both are sometimes inconsistent; and both cohere with other epistemic practices. To my mind the argument that there is no noncircular demonstration of the reliability of Mystical Perception makes a good case for treating it as a fundamental principle—in Alston’s epistemic system.

Seidel disagrees (in response to Kusch (draft)). As Seidel has it, mystical perception and sensory perception are both *instances of perception*; and hence the principle of Instance applies, and rules out the option of treating Mystical Perception as fundamental (2014, p. 167). I am not convinced. It is true of course that in some sense mystical perception has always been modelled on sensory perception. That is after all why we call mystical perception “mystical *perception*”. But it is not obvious to me that we should take our epistemic guidance from such vague analogies. It also is not clear to me how we should think of perception once we have abstracted from both the “mystical” and the “sensory”. In any case, I cannot see why these considerations should be weightier than Alston’s argument to the effect that mystical perception has no noncircular demonstration of its reliability.

Can Seidel’s argument be improved? Rather than saying that Mystical Perception and Sensory Perception are instances of Perception why not say that Mystical Perception and Sensory Perception are instances of a principle called “Seeming”:

(Seeming) If it seems to S that p, and circumstantial conditions D obtain, then S is prima facie justified in believing that p.

It obviously is right to say that Mystical Perception and Sensory Perception (as well as some other principles) can be construed as instances of Seeming. But I am not convinced that this fact tells against the possibility of fundamentally different epistemic systems. The problem is that if the principles common to different epistemic systems become too abstract, too thin, then it is no longer plausible to assume that the common principles prevent the respective epistemic systems from being, intuitively, fundamentally different.

Does Seeming rule out epistemic relativism? One might think so on the following grounds. Epistemic principles differ in what they regard as appropriate conditions for a seeming to confer justification. These differences trace back to factual beliefs about when seemings track the truth. These beliefs can be tested. Moreover, if two incompatible principles (belonging to two different epistemic systems) involve contradictory beliefs about which seemings are truth-tracking, what sense can be made of the relativist’s claim that the two principle could be equally valid?

To answer this worry the epistemic relativist needs to insist again that the testing of factual beliefs does never happen in isolation but only against the background of specific epistemic systems. Does the Poison Oracle of the Azande—I mean the real practice rather than the thin and abstract principle Boghossian formulates—track the truth? That depends on what we mean by “truth” and what we mean by “tracking”. Moreover, remember that Alston argues that neither mystical nor sensory perception have noncircular demonstrations of their reliability. If Alston is right, then the fact that both are instances of seeming does not show that they can be tested and compared in a neutral way.

My second objection to Seidel is based on the idea that the justification for a given source need not all come from *outside of* the source. Initially, Bellarmine may well have justified Revelation in terms of norms and standards that he shares with me. But he may then have gone further: he may have found further evidence for Revelation from reading the Bible. (Maybe the Bible told him to treat Revelation as a fundamental principle). This additional evidence is accessible only to him, not to me. After all, I do not trust this source of evidence. This additional evidence may have led Bellarmine to boost the standing of Revelation to a position as strong as any fundamental principle. This suggestion is again inspired by Wittgenstein’s discussion of certainties. Wittgenstein argues that while initially we might have adopted a belief B on the strength of empirical evidence E, we may later have boosted B to a high epistemic status that—at least under normal conditions—cannot be challenged by new evidence E’. We are more inclined to reject E’ than to reject B.

Objection number three continues with the Wittgensteinian theme. Boghossian and Seidel employ a strict separation of norms and beliefs, arguing that a difference merely in belief is not enough for a fundamental difference of epistemic systems.

As already mentioned, in light of *On Certainty* and the “hinge epistemology” that follows its lead, this distinction is not so clear: “certainties” cut across this distinction. Fundamental beliefs of the certainty variety are no less central to epistemic systems than are norms of the kind cited above.

My fourth objection is directed at the alleged intuitiveness of Derive and Instance. I for one do not have the intuitions that it is the difference in at least one fundamental norm that makes two epistemic systems “genuine alternatives”, or that Instance or Derive adequately capture our assessments of how different two epistemic systems might be. Intuitions to one side, I also suspect that Boghossian’s and Seidel’s criteria do not help to make sense of cases that have motivated epistemic relativism with respect to some important junctures in the history of science. Think for instance of the Chemical Revolution, a case that has recently received a detailed relativist reconstruction (Chang 2012). What would be the fundamental (underived) general epistemic principle on which Priestley and Lavoisier disagreed?

Finally, and in light of this last comment, it seems to me that we need better criteria for judging that two epistemic systems differ in a principled and intractable way.

One promising suggestion it seems to me is to focus on how difficult or how easily imaginable it is to “go over” from our system to the other system—that is, whether we have a “real” or a “notional” confrontation. Do we need a wholesale “conversion” or is it possible to imagine compelling arguments that can force the advocate of one epistemic system to adopt the other? A string of philosophers have suggested that we have fundamentally different systems when a conversion is needed (Wittgenstein 1969; Kuhn 1970; Williams 1981; Van Fraassen 2002).

## Summary

In this paper I have sought to make a contribution to one of the issues that together make up the topic “objectivity in science”. I have discussed one important element in epistemic relativism, No-metajustification, that is, the idea that it is impossible to show in a neutral, non-question-begging, way that one epistemic system is epistemically superior to (all) others. I have focused on two attempts to attack this idea: one of these attempts centred on the relativist uses of the Problem of the Criterion, the other on the claim that there are genuine alternative epistemic systems.

I have tried to show that the relativist’s use of the Problem of the Criterion must take the form of a diagnostic anti-scepticism, and that—in order to avoid falling into scepticism—the relativist must operate with two perspectives. I have also attempted to show that the existing arguments against the possibility of genuine alternative epistemic systems are not compelling.

I have taken my starting point from authors—Sankey, Seidel, Boghossian—who argue against No-metajustification. This is based on the conviction that the best way to make progress in making sense of relativism is to try to dismantle, one by one, the myriad of prima facie compelling arguments against it. This paper is only a very small part of this broader project.
